# Effect of Temperature and Relative Humidity on CO_2_ Adsorption Performance of Biomass-Derived Aerogels

**DOI:** 10.3390/polym17172375

**Published:** 2025-08-31

**Authors:** Zujin Bai, Shuyao Ren, Jun Deng, Chang Su, Furu Kang, Yifan Zhang

**Affiliations:** 1 School of Safety Science and Engineering, Xi’an University of Science and Technology (XUST), Xi’an 710054, China; baizj2022@xust.edu.cn (Z.B.); 24220214076@stu.xust.edu.cn (S.R.); dengj00518@xust.edu.cn (J.D.); kangfur0@xust.edu.cn (F.K.); zhanyf@stu.xust.edu.cn (Y.Z.); 2 Key Laboratory of Mine Exploitation and Disaster Prevention in Western China (XUST), Ministry of Education, Xi’an 710054, China

**Keywords:** biomass aerogel, adsorption characteristics, carbon dioxide, synergy effect, solidification

## Abstract

The safe and efficient capture of CO_2_ in confined environments such as coal mine goafs remains a significant challenge, posing both environmental and safety risks. To address this issue, this study developed a novel biomass-based aerogel adsorbent using CNF-C and CS through sol–gel synthesis and freeze-drying. A series of composite aerogels with varying mass ratios were systematically characterized by SEM, BET, FTIR, and TG-DSC to analyze their microstructure, specific surface area, pore characteristics, chemical properties, and thermal stability. A constant temperature and humidity experimental setup was specially designed to explore the effects of various temperatures, humidity, and material ratios on CO_2_ adsorption performance. FTIR analysis confirmed that -NH_2_ served as the primary adsorption site, with its density increasing with higher chitosan content. The 1:3 ratio exhibited the optimal specific surface area (7.05 m^2^/g) and thermal stability, withstanding temperatures up to 350.0 °C, while the 1:1 ratio demonstrated the highest porosity (80.74%). Adsorption experiments indicated that 35.0 °C and 50% humidity were the optimal conditions, under which the 1:2 ratio biomass aerogel achieved an 18% increase in CO_2_ adsorption capacity compared to room temperature. The sample with a 1:1 high cellulose ratio is primarily dominated by physical adsorption, making its performance susceptible to environmental fluctuations. The sample with a 1:3 high chitosan ratio is predominantly governed by chemical adsorption, exhibiting more stable adsorption characteristics. The 1:2 ratio achieved the best balance under 35.0 °C and 50% humidity. The biomass aerogel synergistically combined physical barriers from its three-dimensional network structure and chemical adsorption via active functional groups, enabling efficient CO_2_ capture and stable sequestration. This study demonstrates the feasibility of biomass-derived aerogels for CO_2_ adsorption under complex conditions and provides new insights into the design of sustainable materials for environmental remediation and carbon reduction applications.

## 1. Introduction

In the global energy consumption structure, coal remains a critical component as a traditional fossil fuel, accounting for 31% of primary energy consumption. As the largest producer and consumer of coal in the world, China still relies heavily on this resource, with coal constituting 55.3% of its primary energy mix [1]. This structure of coal as the main body of energy not only supports economic development but also brings severe environmental challenges. In the recent 20 years, the global cumulative emissions of carbon dioxide from fossil fuel combustion are about 51.0 billion tons, of which coal contributes 53% [2]. The spontaneous combustion of coal in China accounts for 85–90% of the total number of mine fires [3], causing nearly 11 million tons of coal losses annually, direct economic losses of CNY 2.5 billion, and the emission of 19.0 million tons of harmful gases such as CO_2_ [4]. And CO_2_ accumulates in mines with limited ventilation, especially in enclosed areas, which poses a threat to the safety of miners [5]. This not only threatens the safety of miners but also exacerbates the greenhouse effect. So, there is an urgent need to develop new prevention and control technologies that balance safety and environmental protection.

The current reduction of CO_2_ emissions mainly faces three technological bottlenecks [6,7,8,9]. The space for improving the efficiency of fossil fuels is limited and high cost. Renewable energy is hardly able to replace the dominant position of fossil fuels in the short term. The scale of CO_2_ conversion and utilization is limited, and the storage time is short. In contrast, directly storing the generated CO_2_ is more feasible and an optimal choice for achieving effective emission reduction. As a potential CO_2_ storage site, the coal mine goaf creates a huge underground space that provides a natural container for CO_2_ storage through the gaps and fracture networks formed by rock collapse [10].

At present, the storage of CO_2_ in coal mine goaf mainly adopts two technical routes: physical storage and chemical storage. Among them, chemical mineralization storage has become a research focus due to its permanence and safety [11,12]. This technology achieves long-term storage by promoting chemical reactions between CO_2_ and minerals to form stable carbonate compounds, with the key being to solve the diffusion, dissolution, and mass transfer problems of CO_2_ in the reservoir. The innovative storage scheme proposed by the team of academician Wang Shuangming [13] combines deep geological storage, industrial transformation storage, and surface ecological storage to store CO_2_, providing an important technical path for CO_2_ storage in coal mine goaf. These achievements once again prove that CO_2_ solidification in coal mines is a feasible and low-cost means and will definitely be favored by more scholars. Cellulose–chitosan composite aerogels have emerged as ideal functional materials due to their unique synergistic effects and exceptional performance [14]. As the two most abundant biopolymers in nature, cellulose and chitosan possess notable advantages, including renewability, biodegradability, and environmental friendliness [15]. Through composite crosslinking, cellulose provides a rigid framework that enhances mechanical strength, while the amino groups of chitosan form hydrogen bonds and covalent crosslinks with the hydroxyl groups of cellulose. This synergistic interaction endows the composite aerogel with a porosity exceeding 90%, a significantly increased specific surface area, and controllable pore size distribution [16]. This unique structure provides abundant active sites, endowing the material with exceptional adsorption capacity for CO_2_, heavy metals, and organic pollutants [17]. Furthermore, through modification strategies such as carboxylation, hydrophobization, or nanoparticle incorporation, the properties of the material can be precisely tailored, demonstrating broad application potential in environmental remediation, biomedical engineering, and energy storage [18,19,20,21]. The synergistic interaction between cellulose and chitosan not only enhances the mechanical properties and stability of material but also enables functional versatility, establishing it as one of the most promising biomass-based functional materials currently available [22].

The materials predominantly applied for CO_2_ adsorption research include inorganic SiO_2_ aerogels, organic cellulose aerogels, polyimide aerogels, and carbon aerogels, along with various composite aerogels [23,24,25,26]. Enriquet et al. integrated solvothermal synthesis with freeze-casting techniques, employing polyamides of different generations to modify graphene oxide. Through systematic optimization of synthesis conditions, they precisely controlled reaction temperatures and adjusted material ratios. The study revealed that G7-PAMAM-modified aerogels synthesized under conditions of low percentage, high solvothermal temperatures, and liquid nitrogen freezing exhibited optimal CO_2_ adsorption performance [27,28]. The research team led by Pruna developed ethylenediamine-modified graphene oxide aerogels via a one-pot hydrothermal method, with particular emphasis on investigating the effects of oxidation conditions and graphite precursors on the performance of the material. The result demonstrated that expanded graphite-derived aerogels achieved a CO_2_ adsorption capacity of 1.18 mmol/g at 1.0 bar and 298.0 K, along with significantly enhanced surface utilization efficiency [29]. Almahdi et al. developed a composite system combining SiO_2_ aerogel with foam, systematically evaluating the CO_2_ absorption performance, oil displacement, and storage efficiency of the aerogel nanofluid under varying concentrations and gas flow rates. The results show that the composite system appears to have a significantly higher absorption CO_2_ capacity than traditional fluids, and the CO_2_-nanofluid displacement and sequestration rate can reach 60.8%. Moreover, it effectively suppressed gas channeling, showing promising potential for practical applications [30]. Yu et al. fabricated PAMAM-modified graphene oxide aerogels through a hydrothermal-freeze casting method, incorporating carbon nanotubes with precisely controlled ratios. The result confirmed that the aerogel achieved a CO_2_ adsorption capacity of 2.23 mmol/g when the PAMAM to CNT ratio was maintained at 0.6 mg per 0.12 mL. The introduction of CNTs was found to significantly enhance the functionalization degree of material [31]. Based on the above situation, it can be found that traditional aerogel CO_2_ adsorption materials have the problems of low capacity, poor stability, and high cost.

Therefore, to address the aforementioned issues, this work presents an innovative design of multifunctional cellulose and chitosan composite aerogels, with systematic investigation of the key factors governing their CO_2_ adsorption performance and underlying mechanisms. The research first prepared a series of aerogel samples by controlling the cellulose-to-chitosan ratio through sol–gel processing followed by freeze-drying. Comprehensive characterization was performed by conducting SEM, BET, FTIR, and TG-DSC to analyze morphological features, pore structures, functional group distributions, and thermal stability. Finally, the work also investigated the effects of various environmental conditions on the CO_2_ adsorption performance of aerogels. This work demonstrates distinctive novelties: utilizing fully biomass-derived, low-cost cellulose/chitosan to avoid non-renewable precursors; systematically investigating composition-dependent structure–property relationships; evaluating performance under practical coal mine goaf conditions; and elucidating physisorption-chemisorption synergism. This research will provide novel insights for developing high-efficiency stable CO_2_ adsorbent materials and offers significant guidance for implementing carbon sequestration in coal mine goaf areas.

## 2. Materials and Methods

### 2.1. Materials and Instruments

Cellulose (CNF-C, GR), chitosan (CS, GR), polyethylene glycol diglycidyl ether (PEGDGE, AR), and acetic acid (HAc, AR) were purchased from Rohn manufacturers.

The experimental instruments employed included a field emission scanning electron microscope (FEI Quanta 450 with EDAX E250x-max50, Hillsboro, OR, USA), a specific surface area and pore size analyzer (3H-2000PS1, Beishide Instrument Technology, Beijing, China), a Fourier transform infrared spectrometer (Perkin-Elmer Spectrum GX, Norwalk, CT, USA), and a simultaneous thermal analyzer (TGA/DSC, Mettler Toledo, Zurich, Switzerland).

### 2.2. Aerogel Preparation

The first is the preparation of the CNF-C solution and CS solution. A 1 wt% CNF-C suspension was prepared by dispersing 1 g of CNF-C in 99.0 g of deionized water under continuous stirring using a magnetic stirrer for 12 h at room temperature. A 2 wt% CS solution was obtained by dissolving 4 g of chitosan in 194.0 g of deionized water with 2.0 g of acetic acid solution, followed by complete dissolution using magnetic stirring. A 10 wt% PEGDGE solution was prepared by mixing 5.0 g of PEGDGE with 45.0 g of deionized water under vigorous stirring. The aerogel was synthesized via the sol–gel method by mixing predetermined quantities of 1 wt% CNF-C solution and 2 wt% CS solution under homogeneous stirring using a magnetic heating stirrer. Subsequently, 3–4 mL of 10% PEGDGE solution was added as a crosslinking agent to form the final homogeneous mixture. The mass ratio of PEGDGE/(CNF CS) is 1:10.

**Figure 1:**
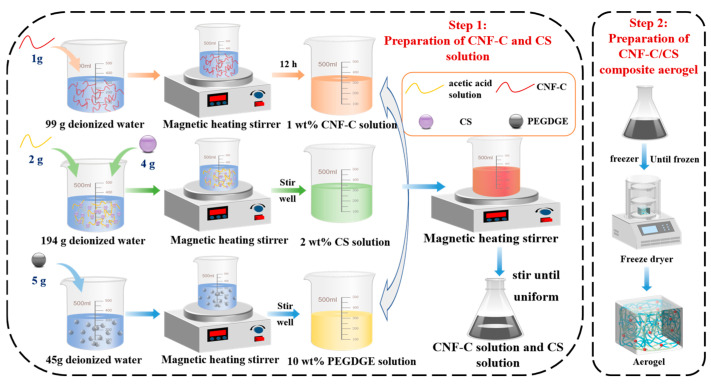
Schematic diagram of the biomass aerogel preparation process.

The second is the preparation of CNF-C/CS composite biomass aerogel. The mixed solution was first placed in a freezing chamber until complete solidification was achieved. The frozen sample was then transferred to a freeze dryer (condenser temperature < −80 °C) and lyophilized for 72 h under a vacuum pressure below 10.0 Pa to obtain the CNF-C/CS composite biomass aerogel. Five cellulose/chitosan biomass aerogel formulations with varying CNF-C to CS mass ratios: 1:1, 1:2, 1:3, 2:3, and 2:5 will be specially developed and explored. This selection covers a broad spectrum from cellulose-rich to chitosan-dominated composites, enabling a systematic study of the effect of composition on the aerogel properties. The preparation workflow is detailed and illustrated in Figure 1.

### 2.3. Physicochemical Characterization of Biomass Aerogels

(1) Fourier transform infrared spectroscopy (FTIR)

Five types of samples were characterized by conducting Fourier transform infrared spectroscopy (FTIR). Each sample is thoroughly mixed, ground, and pressed into transparent particles for spectral analysis. FTIR measurements were performed at 21 ± 5.0 °C with a relative humidity of <65% in a spectral range of 400.0–4000.0 cm^−1^ with a resolution better than 0.09 cm^−1^.

(2) Microstructural morphology testing

The biomass aerogel samples were examined by employing scanning electron microscopy (SEM) to evaluate morphological characteristics. All specimens were sputter-coated with gold for 120 s, adopting an ion sputtering system to enhance surface conductivity. The coated samples were then mounted on standard SEM stubs using conductive adhesive. SEM observations were conducted in high vacuum mode with an accelerating voltage of 20.0 kV and a scanning rate of 0.3 Hz. Images were acquired at various magnifications to characterize the surface morphology of the modified biomass aerogels.

(3) Specific surface area measurement (BET)

The specific surface area and pore size distribution of the composite biomass aerogels were characterized employing nitrogen adsorption–desorption measurements with a full-pore analysis mode. These structural parameters are critical for understanding gas adsorption behavior, and the observed CO_2_ adsorption trends will be analyzed in relation to these surface areas and pore structure characteristics. The degassing time is 3 h, and the degassing temperature is 100.0 °C. This degassing temperature was specifically selected to thoroughly remove adsorbed moisture and gases while preventing the thermal degradation of the temperature-sensitive chitosan component. Nitrogen adsorption isotherms were obtained and subsequently analyzed employing the BET method for surface area determination and the Barrett–Joyner–Halenda (BJH) method for pore size distribution evaluation.

(4) Thermal stability testing (TG-DSC)

The biomass aerogel samples were thoroughly dried in a vacuum oven to remove residual moisture and impurities. The dried materials were then ground into fine powder using an agate mortar to ensure homogeneous heat transfer during thermal analysis. The powdered samples were placed in alumina crucibles and analyzed under a nitrogen atmosphere. The temperature was programmed to increase from 25.0 °C to 800.0 °C at a constant heating rate of 10.0 °C/min, while simultaneously recording mass loss and differential thermal data.

(5) Porosity (*ε*) cellulose and chitosan of biomass aerogels

The porosity was calculated according to Equations (1) and (2) [32].

(1) $$\varepsilon =\left(1-\frac{\rho_{a}}{\rho_{t}}\right)\times \%$$

where the *ρ_a_* denotes the apparent density of the cellulose/chitosan biomass aerogel, while *ρ_t_* represents the skeletal density of the material.

(2) $$\frac{1}{\rho_{t}}=\sum_{k=0}^{n}\left(\frac{\omega_{i}}{\rho_{i}}\right)$$

where *ω_i_* represents the mass fraction of the component, and *ρ_i_* denotes the true density of the component.

### 2.4. CO_2_ Adsorption of CNF-C and CS Biomass Aerogels Testing

#### 2.4.1. Experimental Setup

First, a sealed transparent container was constructed with temperature and humidity sensors mounted on the inner walls. The container was equipped with an adjustable air intake channel and multiple sampling ports. Inside the container, a temperature controller and a humidity controller were installed to create a simple constant temperature and humidity chamber. This setup provided the experimental conditions required to test the CO_2_ absorption performance of biomass aerogels under varying temperature and humidity levels. Next, the five prepared cellulose/chitosan biomass aerogel samples with different ratios underwent degassing and dehydration pretreatment. This step ensured that residual moisture and trapped gases in the pores did not interfere with the CO_2_ absorption capacity of biomass aerogels. Then, each sample in 1 g portions was prepared for subsequent testing.

#### 2.4.2. Experimental Procedure

All biomass aerogel samples to be applied in the experiment were subjected to freeze-drying pretreatment. After 12 h, once residual gases and moisture within the biomass aerogels were completely removed, CO_2_ was introduced into the chamber to purge any residual gases, and a CO_2_ detector was used to monitor the process. A controlled flow of clean air was then slowly introduced through the inlet to stabilize the CO_2_ concentration at 50 vol%, achieving the desired gas environment for the experiment. After establishing the controlled gas atmosphere, a controlled variable approach was employed. The temperature controller and humidity controller were used to regulate the temperature at 25.0, 30.0, 35.0, 40.0, and 45.0 °C. The humidity was 10%, 30%, 50%, 70%, and 90%, respectively. The special selection of various temperatures and humidity takes into account the real environment of the coal mine goaf. The temperature and humidity were maintained at fixed levels. Four partition plates were inserted to enable simultaneous testing of the five types of biomass aerogel samples with varying ratios. The CO_2_ concentration was measured hourly until it stabilized within the experimental environment.

## 3. Results and Discussion

### 3.1. Structural Characterization and Performance of Biomass Aerogels

#### 3.1.1. FTIR Analysis

**Figure 2:**
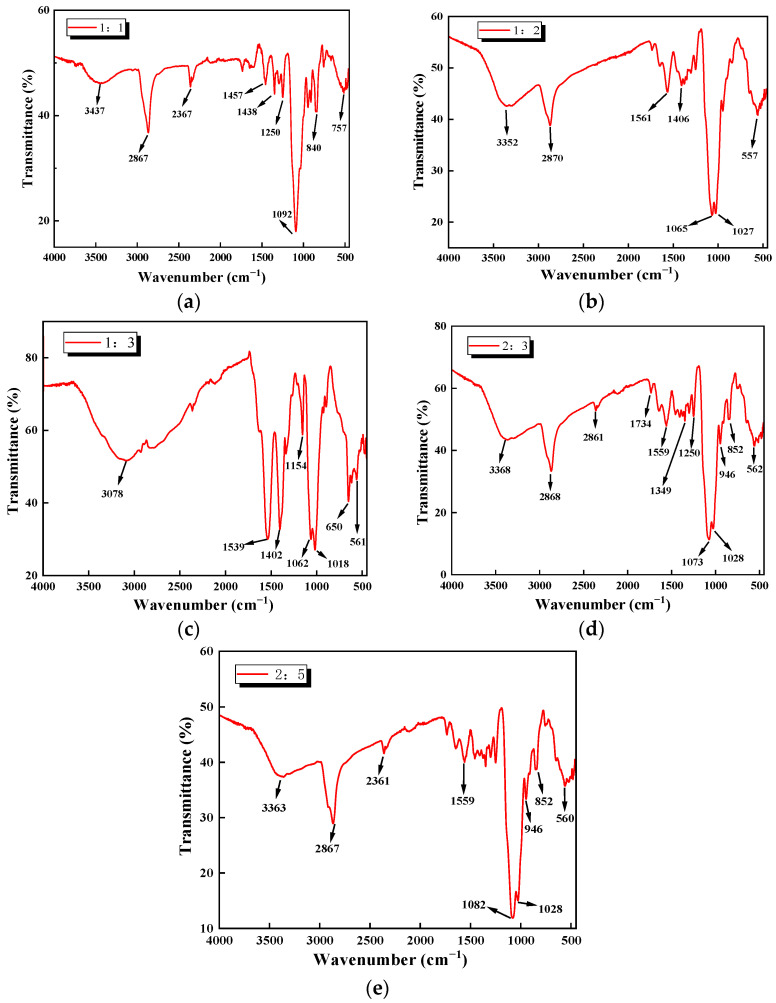
Infrared spectral curves at five types of biomass aerogels. (**a**) Sample at a ratio of 1:1; (**b**) Sample at a ratio of 1:2; (**c**) Sample at a ratio of 1:3; (**d**) Sample at a ratio of 2:3; (**e**) Sample at a ratio of 2:5.

Figure 2 presents the FTIR spectra of five types of biomass aerogel samples with different composition ratios. As observed, all five samples exhibit characteristic absorption peaks in the ranges of 3500–2800 cm^−1^, 700–500 cm^−1^, and 1200–1000 cm^−1^. By identifying and assigning these characteristic peaks, the functional groups present in each sample were determined based on their corresponding absorption band positions, as summarized in Table 1. Comparative analysis suggests that all five types of biomass aerogel compositions contain the functional groups -NH_2_, C-H, C-C, and C-X. These different kinds of functional groups indicate that the targeted composite biomass aerogel was successfully fabricated in this work. Notably, the 1:1, 1:3, and 2:3 ratio samples exhibit additional functional groups such as -OH, =C-H, and C=O. The presence of these extra peaks suggests the PEGDGE or acetic acid residues within the aerogel network, which is a common phenomenon in sol–gel derived systems. A more definitive assignment would require comparative analysis with control spectra of the individual pure components, which is a limitation of this current study and will be considered in future work. It is qualitatively observed that the relative intensity of the characteristic absorption band associated with N–H bending (around 1550–1650 cm^−1^) appears to strengthen with increasing chitosan content, which can be primarily attributed to the increased overall concentration of chitosan-derived amino and amide groups in the composite. The higher the chitosan fraction, the greater the density of these nitrogen-containing functional groups available to contribute to the N–H bending vibration signal, suggesting a higher relative presence of amino groups in these formulations. Furthermore, these abundant amino functional groups provide promising active sites for biomass aerogel to absorb CO_2_, although a direct quantitative correlation between FTIR peak intensity and adsorption capacity is beyond the scope of this qualitative analysis.

#### 3.1.2. Microstructure and Morphology Analysis

**Figure 3:**
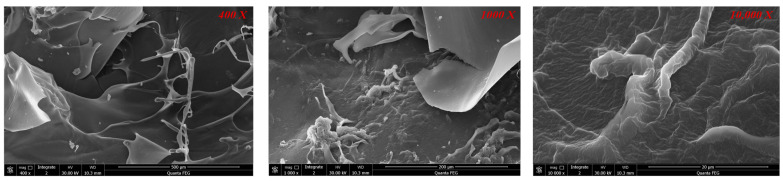
SEM images of biomass aerogel at ratio of 1:1 under different magnifications.

The SEM images of biomass aerogel samples with different composition ratios are presented in Figure 3, take sample 1:1 as an example. The 400× magnified SEM images reveal that all samples exhibit distinct porous structures. Notably, as the chitosan content increases, the pore size of the biomass aerogels gradually decreases. This phenomenon can be attributed to the strong viscosity of chitosan; higher chitosan content tends to clog the porous structure during gel formation. In contrast, samples with higher cellulose content display more pronounced porous networks. This result is due to the support of cellulose, and the higher the cellulose content, the more obvious the support frame in the SEM image of the sample.

Analysis of the 1000× magnified SEM images reveals more pronounced effects of chitosan on the pore structure of biomass aerogel samples. The SEM images of low-chitosan-content biomass aerogels exhibit rough surfaces with distinct protrusions, while appropriate chitosan incorporation results in smoother surfaces with reduced porosity. At 10,000× magnification, the three-dimensional cellulose framework becomes clearly visible in high-cellulose-content biomass aerogels, accompanied by observable surface roughness. This structural feature gradually diminishes with increasing chitosan content, as chitosan progressively fills the porous network and smoothens the surface. There is an interesting phenomenon that when the content of chitosan continues to rise, it can be found that the surface of the biomass aerogel appears as grid-like images and some three-dimensional structures collapse. This phenomenon likely results from excessive crosslinking induced by surplus chitosan, causing pore wall thickening and rigidity reduction. Notably, the 1:3 ratio sample demonstrates the most severe structural collapse, while other composition ratios maintain their original structural integrity.

#### 3.1.3. BET Analysis of Biomass Aerogels

**Figure 4:**
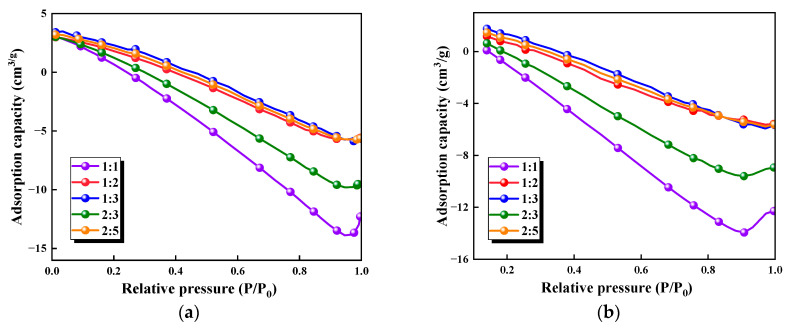
Adsorption and desorption curves of five types of biomass aerogels. (**a**) Adsorption curves; (**b**) Desorption curves.

The nitrogen adsorption–desorption isotherms of the five types of biomass aerogel samples with different composition ratios are presented in Figure 4. In the evaluation of CO_2_ adsorption performance for the biomass aerogels, both specific surface area and pore volume serve as critical parameters, as summarized in Table 2. Analysis of the BET specific surface area data reveals that the 1:1 ratio biomass aerogel exhibits a surface area of 2.2568 m^2^/g, while the 1:3 ratio sample shows a significantly higher value of 7.0515 m^2^/g. This demonstrates a clear trend of increasing specific surface area with higher chitosan content. This can reasonably suggest that elevating the chitosan proportion effectively enhances the surface area of biomass aerogels.

A notable inverse correlation exists between specific surface area and pore volume. The 1:3 ratio sample exhibits the highest surface area (7.05 m^2^/g) yet the lowest pore volume (0.010 cm^3^/g), indicating a fundamental shift in pore architecture toward microporosity. This suggests that increased chitosan content promotes the formation of narrow micropores (<2 nm), which contribute substantially to surface area but minimally to total pore volume. While such microporous structures enhance gas adsorption potential through strengthened gas–solid interactions, the absence of detailed pore size distribution data precludes definitive mechanistic interpretation of diffusion and kinetics, warranting future investigation.

Further examination of the t-Plot pore volume data, which reflects the total internal pore volume, shows distinct variations among different composition ratios. Notably, the relationship between pore volume, surface area, and adsorption performance appears nonlinear, indicating the involvement of additional factors, such as pore structure characteristics and pore size distribution. For instance, while the 1:1 ratio biomass aerogel possesses a relatively large pore volume, its smaller surface area correlates with poorer adsorption performance. In contrast, the 1:3 ratio sample combines both high surface area and large pore volume, resulting in superior adsorption capacity. These observations collectively demonstrate that the adsorption process in biomass aerogels involves complex interactions among multiple structural parameters.

#### 3.1.4. Thermal Stability Analysis

**Figure 5:**
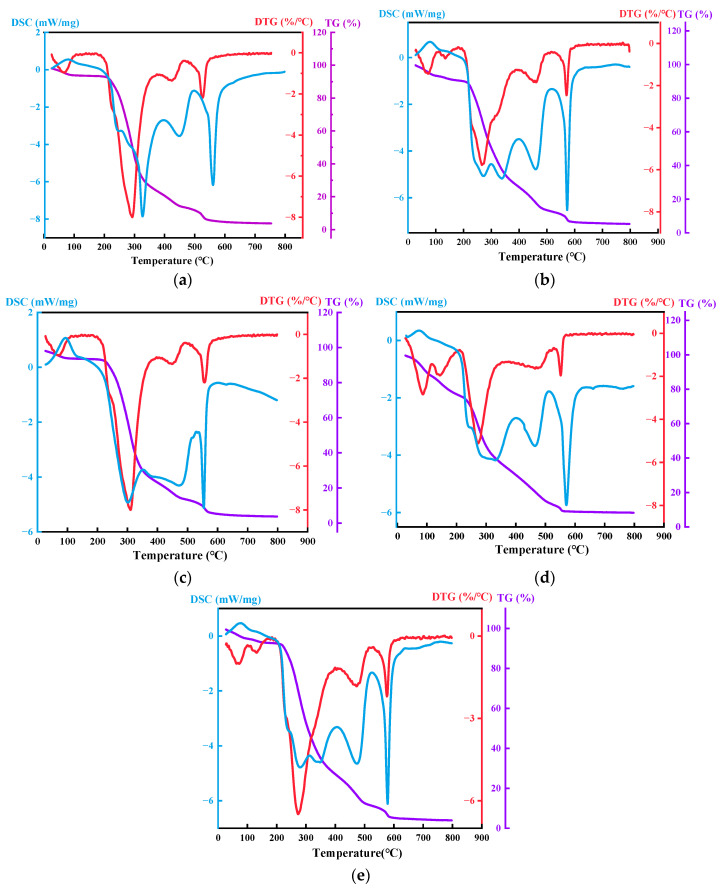
TG-DTG-DSC curves of five ratios of biomass aerogels. (**a**) Sample at a ratio of 1:1; (**b**) Sample at a ratio of 1:2; (**c**) Sample at a ratio of 1:3; (**d**) Sample at a ratio of 2:3; (**e**) Sample at a ratio of 2:5.

The TG/DTG/DSC curves of the five types of samples are presented in Figure 5. All samples showed minimal mass loss during the temperature range of 0–220.0 °C. Notably, the T_5_% varied with the composition. The 1:3 ratio aerogel exhibited the highest onset temperature at approximately 245.0 °C, indicating the widest stable temperature range. In contrast, the 1:1 ratio sample began to degrade at around 220.0 °C. The onset temperatures for the 2:5, 1:2, and 2:3 ratios fell between these two extremes, demonstrating that thermal stability can be tuned by the CNF-C to CS ratio. However, the 1:3 ratio sample exhibited relatively higher mass loss, which may be attributed to the evaporation of residual moisture or volatile impurities within the sample. There exists a significant mass loss that occurred for all samples as temperature increased under 220.0–350.0 °C. The cellulose/chitosan biomass aerogel with a 1:1 ratio demonstrated the highest mass loss, followed by the 2:5, 1:2, and 2:3 ratios. Notably, the 1:3 sample displayed superior thermal stability in this temperature range. The mass change amplitude decreased substantially for all samples at temperatures of 350.0–560.0 °C. The 2:5 ratio sample showed the highest mass loss in this stage, followed by 1:3 and 1:2 ratios. The 1:1 and 2:3 samples exhibited nearly identical mass loss behavior. At this stage, the biomass aerogel samples had essentially completed crosslink breakage and pyrolysis, losing the functional properties.

In the initial 0–200.0 °C range, all samples exhibited minor fluctuations in DTG curves. As the temperature increased to 200.0–310.0 °C, each of the DTG curves of the sample sequentially reached its minimum value, and before stabilizing at approximately 400.0 °C, the specific temperatures corresponded to the detailed summary in Table 3.

To correlate mass loss with thermal events, TG-DSC analysis reveals coordinated processes: the major mass loss at 220–350 °C corresponds to endothermic peaks near 215–392 °C, indicating polymer backbone scission and cross-link breakdown. Subsequent gradual mass loss (350–560 °C) aligns with endothermic events at 415–507 °C, suggesting deep decomposition and carbonization. Minor fluctuations near 550–600 °C coincide with final endothermic peaks, likely from late-stage carbonization or residue phase changes.

There is a fluctuation in the range of 550.0–600.0 °C, and then it returns to stability again. The thermal decomposition behavior of the materials exhibited significant variations depending on their composition ratios. All samples displayed three to four characteristic endothermic peaks within the temperature range of 215.0 °C to 590.0 °C, corresponding to three distinct thermal decomposition stages, including polymer backbone scission, deep decomposition, and residual carbonization. Under conventional operating temperatures below 350.0 °C, the sample at a ratio of 1:3 with higher chitosan content demonstrated optimal thermal stability, as evidenced by its highest onset decomposition temperature. The measured decomposition enthalpy was 1048.0 J/g for the 1:3 sample, compared to 1943.0 J/g for the least stable 1:1 ratio sample. The higher enthalpy value may indicate a more complex or energy-intensive decomposition pathway for the 1:1 composition. Notably, all samples underwent structural failure due to chemical bond breakage within the critical temperature range of 350.0 °C to 400.0 °C. When temperatures exceeded 550.0 °C, the ratio of 1:1 exhibited superior stability during carbonization compared to the ratio of 1:3. Comprehensive evaluation revealed the following thermal stability ranking among the five types of biomass aerogel formulations from highest to lowest stability: 1:3, 2:5, 1:2, 2:3, and 1:1. These findings provide essential guidance for selecting and applying biomass aerogel materials under different temperature conditions. The superior stability of the 1:3 formulation below 350 °C suggests its potential suitability for applications requiring thermal regeneration processes at moderate temperatures. Conversely, the high-temperature carbonization behavior observed above 550 °C may inform potential uses in high-temperature environments or as a precursor for carbon materials.

#### 3.1.5. Porosity Characteristic Analysis

To calculate the porosity of each ratio of biomass aerogels, it is necessary to determine the apparent density *ρ_a_* and the true density *ρ_t_*. The calculated results for apparent density, true density, and porosity of the biomass aerogel samples are presented in Table 4. It can be reflected from Table 4 that the cellulose/chitosan biomass aerogels with different ratios exhibit significant variations in porosity, indicating distinct pore characteristics among the formulations, which consequently leads to differences in their CO_2_ absorption performance. The porosity of these five proportioned samples in descending order is 1:1, 1:3, 1:2, 2:5, and 2:3. Specifically, the 1:1 ratio biomass aerogel demonstrates the highest porosity of 80.74% among all formulations. In contrast, the 2:3 ratio biomass aerogel appears the lowest porosity of 72.59%, which is 8.15% lower than the 1:1 ratio. Porosity also significantly influences both the CO_2_ absorption capacity and the stability of the biomass aerogel samples. So abundant pore walls in the biomass aerogel provide more loading sites for the active site amino groups. One of the main reasons for biomass aerogel to absorb CO_2_ is to use the active site amino on the pore wall of the gel, which reacts with CO_2_ under the action of water, thus enhancing the absorption efficiency. Therefore, the amount of porosity directly determines the capacity of biomass aerogel to absorb CO_2_.

### 3.2. CO_2_ Adsorption Performance of CNF-C/CS Biomass Aerogels

#### 3.2.1. Effects of Temperature on CO_2_ Adsorption Performance

**Figure 6:**
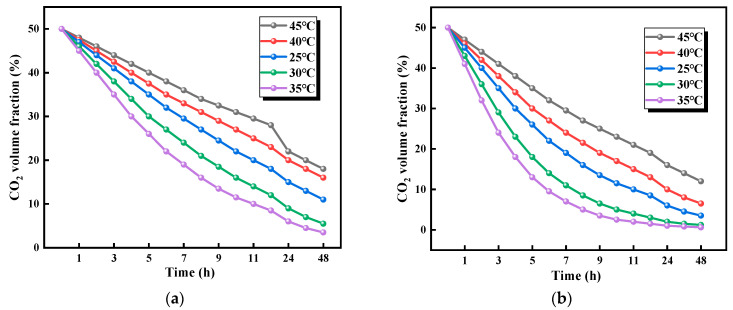

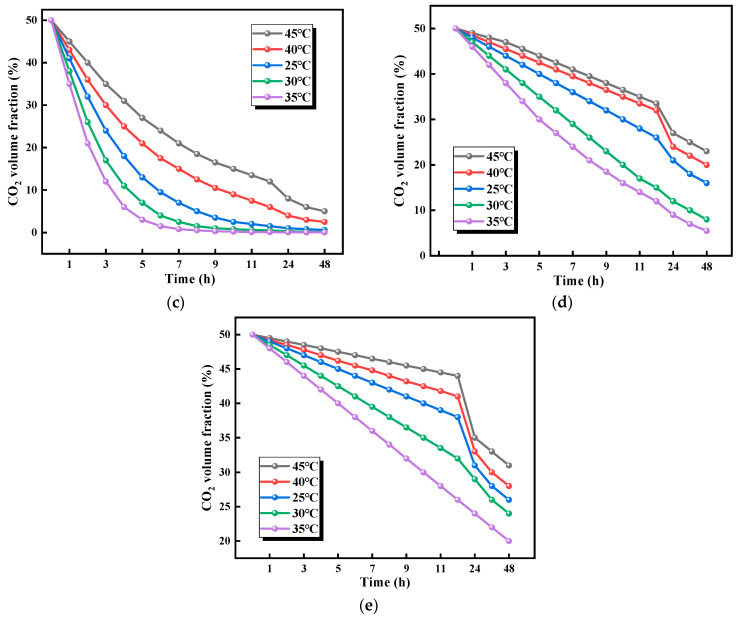
Effect of temperature on CO_2_ absorption performance of biomass aerogel. (**a**) Sample at a ratio of 1:1; (**b**) Sample at a ratio of 1:2; (**c**) Sample at a ratio of 1:3; (**d**) Sample at a ratio of 2:3; (**e**) Sample at a ratio of 2:5.

The effects of temperature on the CO_2_ absorption performance of biomass aerogels are illustrated in Figure 6. The results demonstrate that all five types of biomass aerogel formulations exhibit CO_2_ absorption capabilities. As shown in Figure 6, starting from 25.0 °C with 5.0 °C increments up to 45.0 °C, the CO_2_ absorption efficiency of all five samples shows a positive correlation with temperature. Furthermore, as temperature increases, the absorption curves of different formulations exhibit a more pronounced trend toward stabilization. When the ambient temperature of the 1:1 biomass aerogel sample is 45.0, 40.0, 35.0, 30.0, and 25.0 °C, the remaining CO_2_ in the device is 19%, 16%, 3%, 7%, and 11%, respectively. The optimal CO_2_ absorption performance was achieved at 35.0 °C, while the poorest performance occurred at 45.0 °C, with a 16% improvement in absorption compared to 45.0 °C. Notably, temperature exerted varying effects on the absorption capacity of biomass aerogels with different compositions. For instance, the 1:2 ratio biomass aerogel showed an 18% difference in CO_2_ absorption between 35.0 °C and 45.0 °C, whereas the 1:3 ratio biomass aerogel exhibited only a 5% difference under the same temperature variation.

For all five types of biomass aerogel formulations, 35.0 °C was identified as the optimal temperature for CO_2_ absorption, while 45.0 °C showed the poorest absorption performance. For the 1:1 ratio biomass aerogel sample, while CO_2_ absorption efficiency varies with temperature changes, the fluctuations are relatively moderate. This phenomenon can be attributed to the balanced cellulose-to-chitosan ratio in this formulation. The predominant CO_2_ absorption mechanism involves physical adsorption by cellulose, complemented by minimal chemical absorption through chitosan. This composition accounts for the lower sensitivity of the sample to temperature variations. The 1:2 ratio sample similarly exhibited its optimal CO_2_ absorption at 35.0 °C, while demonstrating significantly poorer performance at 45.0 °C compared to other temperatures. A comprehensive analysis of all formulations leads to a consistent conclusion: 35.0 °C represents the optimal temperature for CO_2_ absorption by biomass aerogel samples within the 25.0–45.0 °C range, while 45.0 °C proves to be the least favorable temperature condition.

The compositional ratio stands as a critical factor that determines adsorption performance. At 25.0 °C, the 1:2 ratio achieved superior adsorption, showing 4% residual CO_2_, which significantly outperformed the 30% residual CO_2_ observed for the 2:5 ratio, representing a 26% difference. This performance advantage remained consistent with 23% and 20% differences measured at 40.0 °C and 45.0 °C, respectively. Particularly noteworthy remains the exceptional environmental adaptability demonstrated by the 1:2 ratio, where the adsorption curves exhibited progressive saturation trends as temperature increased. Although the cellulose-rich 2:5 ratio displayed the poorest performance, its adsorption efficiency still improved with rising temperature.

These results reveal that balanced formulations such as the 1:1 ratio mainly depend on synergistic effects between physical adsorption occurring through cellulose and weak chemical adsorption facilitated by chitosan, leading to relatively moderate temperature sensitivity. No collapse occurred in the cellulose framework, and no deactivation took place in the chitosan active sites across the tested temperature range, confirming excellent thermal stability. These findings provide crucial guidance for optimizing biomass aerogel adsorbent selection under various thermal conditions where 35.0 °C represents the universally optimal temperature, the 1:2 ratio offers the best comprehensive performance, while the chitosan-rich 1:3 ratio proves more suitable for applications experiencing significant temperature fluctuations.

#### 3.2.2. Effects of Humidity on CO_2_ Adsorption Performance

**Figure 7:**
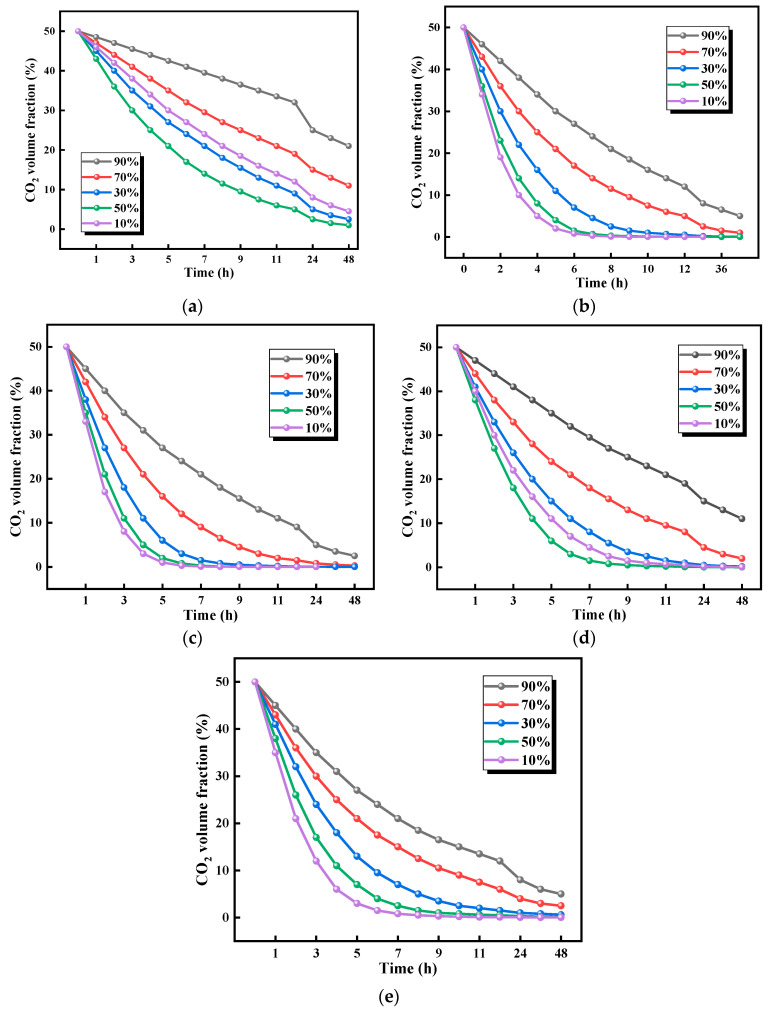
Absorption curves of aerogels with various proportions in five humidity environments. (**a**) Sample at a ratio of 1:1; (**b**) Sample at a ratio of 1:2; (**c**) Sample at a ratio of 1:3; (**d**) Sample at a ratio of 2:3; (**e**) Sample at a ratio of 2:5.

The effects of humidity on the CO_2_ absorption performance of biomass aerogels are presented in Figure 7. When the ratio of biomass aerogel samples with 1:1 is 10%, 30%, 50%, 70%, and 90% in the humidity environment, the residual amount of CO_2_ is 5%, 3%, 2%, 12%, and 23%, respectively. The 1:1 ratio biomass aerogel demonstrates distinct performance variations across different humidity levels, achieving optimal absorption at 50% relative humidity with only 2% residual CO_2_, while showing the poorest performance at 90% humidity with 23% residual CO_2_. Comparative analysis of different formulations shows the 1:2 ratio exhibits a 7% absorption difference between 50% and 90% humidity, whereas the 1:3 ratio shows merely a 3% variation, indicating reduced humidity sensitivity with higher chitosan content.

Within the 10–90% humidity range, 50% relative humidity represents the optimal absorption condition for all biomass aerogel samples, delivering maximum absorption efficiency, while 90% humidity results in the poorest performance. Notably, the 1:2 ratio aerogel demonstrates superior environmental adaptability, maintaining excellent performance across various humidity conditions and retaining relatively high absorption efficiency even under high humidity. Mechanistic analysis indicates that below 50% humidity, all five types of biomass aerogel formulations show improved absorption efficiency with increasing humidity. However, when humidity exceeds 50%, water molecule coverage on the biomass aerogel surface simultaneously obstructs physical adsorption sites on cellulose and interferes with the chemical adsorption activity of chitosan, leading to reduced absorption efficiency. Importantly, no cellulose framework collapse or chitosan active site deactivation was observed throughout the humidity variations, confirming excellent humidity stability. These findings provide crucial guidance for practical applications of biomass aerogel-based CO_2_ adsorption materials in real-world environments.

These results indicate that the compositional ratio of biomass aerogels critically determines their CO_2_ adsorption performance. Under 10% relative humidity, significant variations in residual CO_2_ levels were observed across different biomass aerogel formulations, with the 1:2 ratio exhibiting optimal performance at just 3% residual CO_2_, while the 2:5 ratio showed the poorest performance at 27%, representing a 24% difference. As humidity increased to 30%, the adsorption difference between 1:2 and 2:5 ratios measured 22%, narrowing to 16% at 50% humidity. All five types of biomass aerogel formulations demonstrated measurable CO_2_ adsorption capacities, with the 1:2 ratio consistently delivering superior performance. Although the 1:1 ratio sample showed humidity-dependent variations in adsorption performance, the overall fluctuations remained relatively moderate. Mechanistic analysis reveals that biomass aerogels capture CO_2_ through synergistic physical adsorption by cellulose and chemical adsorption by chitosan. Below 50% humidity, environmental moisture enhances the chemical reaction between chitosan and CO_2_. However, when humidity exceeds 50%, water molecules coat the aerogel surface, simultaneously reducing cellulose’s physical adsorption capacity and obstructing access to chemical adsorption sites, thereby diminishing overall adsorption efficiency.

In summary, within the 10–90% humidity range, 50% relative humidity represents the optimal condition for CO_2_ adsorption by biomass aerogel samples, while 90% humidity yields the poorest performance. The 1:2 ratio biomass aerogel achieves maximum adsorption efficiency at 50% humidity but shows significantly reduced performance at 90% humidity. These findings provide essential guidance for optimizing aerogel-based CO_2_ adsorption materials under varying humidity conditions.

### 3.3. Adsorption Mechanism Analysis

**Figure 8:**
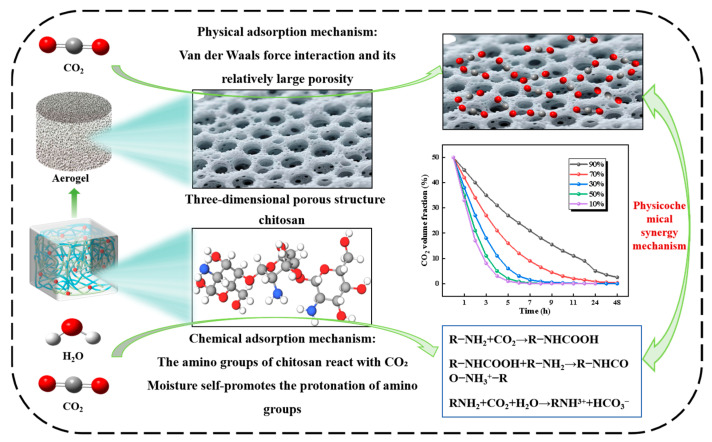
Mechanism diagram of CO_2_ absorption by biomass aerogels.

The cellulose/chitosan-composite biomass aerogel achieves efficient CO_2_ capture through synergistic physical and chemical adsorption mechanisms. The CO_2_ adsorption mechanism is described in Figure 8. The physical adsorption mechanism is manifested through macropore structures that enable van der Waals force interactions, with the substantial porosity providing abundant physical adsorption sites for CO_2_ molecules. The incorporation of increased chitosan content established a crosslinked network, reducing pore wall thickness from 50 to 100 μm (1:1 ratio) to 20–50 μm (1:3 ratio). This structural optimization enhanced pore stability while preserving physical adsorption sites, achieving an optimal balance between adsorption capacity and structural integrity.

The chemical adsorption is proposed to primarily involve the reaction between the -NH_2_ of chitosan and CO_2_ to form carbamates at 50% relative humidity. The reaction is expected to correlate with amino group density, which is qualitatively suggested by a 28% enhancement in peak intensity for the 1:3 ratio sample compared to baseline formulations. Humidity modulation is understood to demonstrate that moderate moisture promotes amino group protonation, while excessive water molecules saturate active sites through hydrogen bonding. Based on established literature, the highest efficiency for carbamate formation is reported to occur at around 50% relative humidity. The reaction formula is:

$$RNH_{2}+CO_{2}+H_{2}O⇌RNH_{3}^{+}+HCO_{3}^{-}$$

The material ratio and environmental conditions significantly affect the adsorption properties. The optimal 1:2 ratio sample achieved an ideal balance between physical and chemical adsorption at 35.0 °C under 50% relative humidity, demonstrating a CO_2_ adsorption capacity of 2.8 mmol/g. It is hypothesized that low humidity facilitates amino group protonation, while humidity exceeding 50% saturates the amino sites with water molecules. Elevated temperatures disrupt amino group configurations, and high humidity blocks pore channels, collectively leading to diminished adsorption performance.

It is important to note that the proposed chemisorption mechanism, while consistent with the observed performance data and well-established literature, requires further validation through advanced characterization techniques for direct chemical confirmation of the adsorption products. Such confirmation remains a valuable direction for future investigation.

## 4. Conclusions

This work successfully prepared CNF-C/CS biomass aerogel samples with different composition ratios (1:1, 1:2, 1:3, 2:3, 2:5). The biomass aerogels were systematically characterized by employing SEM, BET, FTIR, and TG-DSC to investigate microstructure, specific surface area, pore characteristics, chemical structure, thermal stability, and porosity. A controlled-variable approach was specially designed and employed to quantitatively explore the effects of temperature and humidity conditions on CO_2_ absorption performance. Finally, mechanisms of the biomass aerogel system adsorption CO_2_ were comprehensively investigated and elucidated. The detailed conclusion is summarized as follows:

(1) FTIR analysis confirmed that -NH_2_ functional groups on the biomass aerogel surface served as primary active sites for CO_2_ adsorption, with concentration showing positive correlation with chitosan content. BET and SEM characterization revealed increased specific surface area with higher chitosan ratios. Though accompanied by progressive pore densification, excessive chitosan caused structural collapse. The sample with a ratio of 1:3 has the superior thermal stability before 350.0 °C, while the 1:1 ratio exhibited the poorest. The maximum void fraction is 80.74% in the ratio of 1:1, and sample and pore architecture varied substantially across different compositions. These structural characteristics directly governed the capture performance of CO_2_ of biomass aerogel. Current SEM analysis is qualitative; future work should quantitatively correlate image-based morphological features with BET/BJH pore structure parameters.
(2) Within the 25.0–35.0 °C range, CO_2_ adsorption capacity increased with temperature, reaching optimal performance at 35.0 °C. Beyond 35.0 °C, adsorption capacity declined due to thermally induced structural degradation of the material. The optimal adsorption efficiency was achieved at 50% relative humidity, and maximum adsorption efficiency at 50% relative humidity. CO_2_ uptake showed a positive correlation with humidity in the 10–50% relative humidity range but decreased above 50% relative humidity due to competitive occupation of active sites by water molecules. Notably, distinct composition-dependent behaviors were observed. The cellulose-rich 1:1 ratio exhibited predominantly physical adsorption, demonstrating greater sensitivity to temperature and humidity variations. In contrast, the chitosan-rich 1:3 ratio displayed chemical adsorption-dominated behavior with superior environmental stability.
(3) The key finding reveals that the biomass aerogel achieves efficient CO_2_ capture through a synergistic physicochemical mechanism. Physical adsorption relies on the three-dimensional porous structure, where the 80.74% porosity of the 1:1 ratio provides abundant van der Waals adsorption sites. Increased chitosan content constructs a crosslinked network, reducing pore wall thickness to 20–50 μm while significantly enhancing structural rigidity. Chemical adsorption occurs through carbamate formation between the -NH_2_ groups of chitosan and CO_2_ at 50% humidity, with the 1:3 ratio exhibiting 28% higher amino group density. Water molecules demonstrate dual functionality. Moderate humidity promotes amino protonation, while excessive humidity induces competitive hydrogen bonding that inhibits adsorption. This synergistic mechanism optimizes the balance between pore architecture and amino group density, ultimately enhancing CO_2_ adsorption efficiency.

## Figures and Tables

**Table 1:** Types of functional groups contained on the samples.

Cellulose/Chiosan Ratios	Functional Groups
1:1	-NH2, C-H, -C-C-, C-X, -OH
1:2	-NH2, C-H, -C-C-, C-X
1:3	-NH2, C-H, -C-C-, C-X, =C-H
2:3	-NH2, C-H, -C-C-, C-X, C=O
2:5	-NH2, C-H, -C-C-, C-X

**Table 2:** Specific surface area and pore volume of all samples.

Cellulose/Chitosan Ratios	BET Specific Surface Area (m^2^/g)	t-Plot Hole Capacity (cm^3^/g)
1:1	2.2568	0.015006
2:3	3.3319	0.012112
1:2	5.3976	0.010670
2:5	6.1235	0.010603
1:3	7.0515	0.010067

**Table 3:** Temperatures at the lowest point of DTG curves for samples at 200.0–310.0 °C.

Ratios	1:2	1:3	2:5	2:3	1:1
Temperature	266.1 °C	272.5 °C	273.5 °C	281.7 °C	310.1 °C

**Table 4:** Apparent density, true density, and porosity of biomass aerogels.

Cellulose/Chitosan Ratios	*ρ_a_* (g/cm^3^)	*ρ_t_* (g/cm^3^)	ε (%)
1:1	0.26	1.35	80.74
1:2	0.33	1.34	75.37
1:3	0.29	1.32	78.03
2:3	0.37	1.35	72.59
2:5	0.34	1.31	74.04

## Data Availability

Data will be made available upon request.

## Abbreviations

The following abbreviations are used in this manuscript:

AR	Analytical Reagent
GR	Guaranteed Reagent
BET	Specific surface area measurement
HAc	acetic acid
PAMAM	Polyamidoamine
CNF-C	Carboxylated Cellulose Nanofibers
PEGDGE	Polyethylene glycol diglycidyl ether
SEM	Scanning electron microscopy
TG	thermogravimetry
CNT	Carbon Nanotubes
TG-DSC	Thermal stability testing
CS	Chitosan
DTG	Derivative thermogravimetry
DSC	Differential scanning calorimetry
FTIR	Fourier transform infrared spectroscopy
ε	Porosity
